# Role of Annexin A1 in Squamous Cell Lung Cancer Progression

**DOI:** 10.1155/2021/5520832

**Published:** 2021-04-17

**Authors:** Omar Elakad, Yuchan Li, Natascha Gieser, Sha Yao, Stefan Küffer, Marc Hinterthaner, Bernhard C. Danner, Alexander von Hammerstein-Equord, Philipp Ströbel, Hanibal Bohnenberger

**Affiliations:** ^1^Institute of Pathology, University Medical Center, Göttingen, Germany; ^2^Department of Thoracic and Cardiovascular Surgery, University Medical Center, Göttingen, Germany

## Abstract

Lung cancer remains the primary cause of cancer-related death worldwide, and its molecular mechanisms of tumor progression need further characterization to improve the clinical management of affected patients. The role of Annexin A1 (ANXA1) in tumorigenesis and cancer progression in general and especially in lung cancer remains to be controversial and seems to be highly tissue specific and inconsistent among tumor initiation, progression, and metastasis. In the current study, we investigated ANXA1 expression in 81 squamous cell lung cancer (SQCLC), 86 pulmonary adenocarcinoma (AC), and 30 small cell lung cancer (SCLC) patient-derived tissue samples and its prognostic impact on patient's survival. Mechanistically, we analyzed the impact of ANXA1 expression on proliferation and migration of SQCLC cell lines using CRISPR-Cas9 and mammalian overexpression vectors. Strong expression of ANXA1 was significantly correlated to longer overall survival only in SQCLC patients (*P* = 0.019). Overexpression of ANXA1 promoted proliferation in SQCLC cell lines but suppressed their migration, while knockout of ANXA1 promoted cell migration and suppressed proliferation. In conclusion, ANXA1 expression might elongate patients' survival by inhibiting tumor cell migration and subsequent metastasis.

## 1. Introduction

According to the 2020 World Cancer Report, lung cancer continues to cause the grand majority of cancer-related mortality (18%) [1]. Approximately two million new cases are diagnosed each year worldwide [2]. Lung cancer is histologically classified into small cell lung cancer (SCLC) and nonsmall cell lung cancer (NSCLC). NSCLC represents around 85% of all lung cancers and comprises two predominant histological subtypes: adenocarcinoma (AC, 65%) and squamous cell carcinoma (SQCLC, 30%) [3, 4]. Although great progress in revealing the sophisticated genomic landscape of lung cancer has been accomplished, the understanding of the molecular mechanisms of tumor progression is still limited [5–7].

Annexin A1 (ANXA1) is the first member of the phospholipid-binding Annexin superfamily of proteins [8]. The earliest discovered role of ANXA1 was its anti-inflammatory role to suppress neutrophils and modulated monocytes recruitment [9]. Dysregulation of ANXA1 expression has been reported to contribute to the pathophysiology of multiple processes, such as tumorigenesis and auto-inflammatory diseases [10–16].

In tumorigenesis, the expression of ANXA1 was found to be highly tissue and tumor specific. For instance, ANXA1 has been reported to be upregulated in breast cancer, hepatocellular carcinoma, and melanoma while downregulated in gastric cancer, prostate cancer, and oral cancer [17–22]. Like its expression patterns, the role of ANXA1 has been found to be inconsistent among tumor initiation, progression, and metastasis where it has been shown to be not only a tumor suppressor but also a tumor promotor. For example, in breast cancer, ANXA1 tends to inhibit cancer cell proliferation and metastatic potential [23]. However, ANXA1 promotes tumor development by inducing cell migration and invasion with formyl peptide receptors in colon and gastric cancers [16, 24–26]. Furthermore, ANXA1 expression has been reported to vary depending on the grade of differentiation in oral squamous cell carcinoma [27–29].

In the current study, we aimed to elucidate the prognostic impact of ANXA1 in SQCLC patients. Furthermore, we investigated the molecular function of ANXA1 in tumor cell proliferation and migration. Our results revealed a significant correlation between ANXA1 expression and longer overall survival only in SQCLC patients. Knockout and overexpression of ANXA1 in SQCLC cell lines revealed a role in inhibiting cellular migration.

## 2. Material and Methods

### 2.1. Tissue Samples

Lung cancer patient samples were obtained from surgical resections at the University Medical Center of Goettingen (UMG). Collection and use of the samples were approved by the ethics committee at UMG. Informed consent was granted by all patients. All investigations were conducted in conformity to the declaration of Helsinki and institutional, state, and federal guidelines. All investigations were conducted in agreement with the declaration of Helsinki and institutional, state, and federal guidelines.

### 2.2. Immunohistochemistry

Tissue microarrays were prepared and stained as published before [30, 31]. Briefly, tissues were cut into sections of 2 *μ*M, incubated in EnVision Flex target retrieval solution (Dako) at low pH, incubated with primary anti-ANXA1 antibody (dilution: 1 : 50, #011272, Sigma) for 20 minutes at room temperature and secondary antibody (EnVision Flex +, Dako). ANXA1 polyclonal antibody was validated by the Human Protein Atlas (HPA011272) for staining (compared to RNA expression) and distribution in 2 human cell lines and over 40 tissues. The antibody targets N-terminal end of the protein with the following epitope sequence: MAMVSEFLKQAWFIENEEQEYVQTVKSSKGGPGSAVSPYPTFNPSSDVAALHKAIMVKGVDEATIIDILTKRNNAQRQQIKAAYLQETGKPLDETLKKAL [30]. To counterstain tissues, Mayer's hematoxylin stain was used. Samples were analyzed under 100× and 400× of light microscopy. The staining intensity of ANXA1 in the cytoplasm of cancer cells was classified into “zero” for negative expression, “one” for weak expression, and “two” for strong expression.

### 2.3. Cell Culture

Seven SQCLC cell lines were used. H1703 was supplied by AddexBio, LK2 was supplied by JCRB, and H520, HCC15, SKMES-1, H2170, and H226 were supplied by ATCC. Cell lines were cultured under RPMI-1640 growth medium containing 1% penicillin/streptomycin, 1% glutamine, and 10% fetal bovine serum (FBS). The cell line H520-ANXA1, H520-Empty, H1703-ANXA1, H1703-empty, LK2-ANXA1, and LK2-Empty were obtained by transfection with pEGFP-anxA1 (Addgene_107194) or control pEGFP vectors. All transfected cells were grown under RPMI-1640 with 10% FBS, 1% glutamine, 1% penicillin/streptomycin, and neomycin (H520/H1703:1500 *μ*g/mL; Lk2:700 *μ*g/mL, G418, InvivoGen, San Diego, California, USA). Medium containing neomycin for selection was changed twice a week. All cells were passaged every four to five days and kept at 37°C in an atmosphere of 20% oxygen and 5% CO2. For passaging, Trypsin-EDTA was used.

### 2.4. Western Blotting

Cells were rinsed using ice-cold PBS and collected then lysed in RIPA lysis buffer. Equivalent protein samples (15 *μ*g) were separated by 4-15% SDS-PAGE gel (Bio-Rad Laboratories, Inc.) and electro-transferred onto nitrocellulose membrane by trans-blot turbo transfer system (Trans-Blot Bio-Rad Laboratories) then incubated with anti-ANXA1 antibody ((D5V2T) XP®, Cell Signaling). Plus-ECL (PerkinElmer) was used to develop the signals. As a loading control, PARK7 (ab18257, Abcam) was used due to its high consistency [31]. Nitrocellulose membranes were visualized using Fusion Fx7 fluorescence imaging system.

### 2.5. Small Interfering RNA Transfection

For knockdown experiments, ANXA1 siRNA targeting the sequence CATCATTGACATTCTAACTAA (QIAGEN N.V., Venlo, Netherlands) or control siRNA (Allstars, Qiagen, No.1027281) was used at a concentration of 30 nM. Investigation of transfections efficiency of siRNA was performed using AllStars negative siRNA AF-488 (Allstars, Qiagen, No. 1027281). Before transfection, cells were harvested, centrifuged at 1200 rpm for 5 min, counted using Muse® Count & Viability Assay, and seeded at appropriate confluency. siRNA was incubated in serum-free medium containing Hiperfect for 10-15 min at RT and added dropwise to the seeded cells.

### 2.6. CRISPR-Cas9 Knockout

Guide RNA was designed to target the ANXA1 gene (gRNA-1 forward: P-CACCGAGACATTAACAGGGTCTACAGAG, reverse: AAACCTCTGTAGACCCTGTTAATGTCTC). Guide RNAs were cloned into Cas9 plasmid backbone with a reporter fluorescent green protein (PX458 plasmid, Addgene). Successfully transfected cells were sorted into single cells where the gene knockout was validated using western blot and DNA sequencing.

### 2.7. Cell Viability Analysis

The desired number of cells per well was diluted in 100 *μ*L of the appropriate medium. At 24 h intervals, CellTiter 96@ Aqueous One Solution (Promega) was added and incubated at 37°C for 2.5 h. Absorbance measurement was performed at 490 nm using a microplate reader.

### 2.8. Cell Counting

In 24-well plates, 25,000 cells were seeded per well in triplicates. For knockdown experiments, cells were treated with control siRNA and ANXA1 siRNA and then incubated for 120 h at 37°C in an atmosphere of 20% oxygen and 5% CO2. Growth medium of cells was removed and stored until cells were trypsinized and resuspended. Cell counting was performed using Muse® Count & Viability Assay (Merck KGaA, Darmstadt, Germany) according to the manufacturer's protocol.

### 2.9. Scratch Wound Assay

In 6-well plates, cell lines were cultured until 90% confluency. A scratch was introduced to the epithelial layer of the cells using a pipette tip. The diameter of scratch was consistent among all cell lines (10 mm) then scratched cells were washed once. Wound healing was represented by the distance covered by cells from its edge to the wound area compared to control cell lines. The wound closure was monitored with a Canon EOS 650D camera mounted on an inverted microscope. Measurements were taken after scratching (0 h) and at different observation time points with three images taken for each time point. ImageJ was employed to analyze the image and measure the distance using the superimposed grid between the edges of the wound.

### 2.10. Statistical Analysis

Patients' overall survival was correlated to ANXA1 protein expression using Kaplan-Meier analysis. Correlation between different pathological features of lung cancer patients and ANXA1 expression was carried out using Pearson's coefficient, contingency tables, and Chi-square tests. *P* values were determined using Chi-square test (Mantel-Cox) integrated in GraphPad Prism 7.

## 3. Results

### 3.1. Prognostic Impact of ANXA1 Expression in Human Lung Cancer

A cohort of 197 lung cancer patient samples was collected in order to study the prevalence and prognostic value of ANXA1 protein expression among lung cancer patients. Clinical and histopathological characteristics of the patients can be obtained from Supplementary Table 1 and are summarized in Table 1. The cohort included 81, 86, and 30 patient samples diagnosed as SQCLC, AC, and SCLC, respectively. The median age of patients was 67 years (range = 34 − 84). Male patients formed 67% of the cohort. Tumors were diagnosed at different stages according to UICC (7^th^ edition) (80, 59, 46, and 4 patients at stages I, II, III, and IV, respectively). All patients were treated with surgical resection of the tumor without prior neoadjuvant chemotherapy.

**Figure 1:**
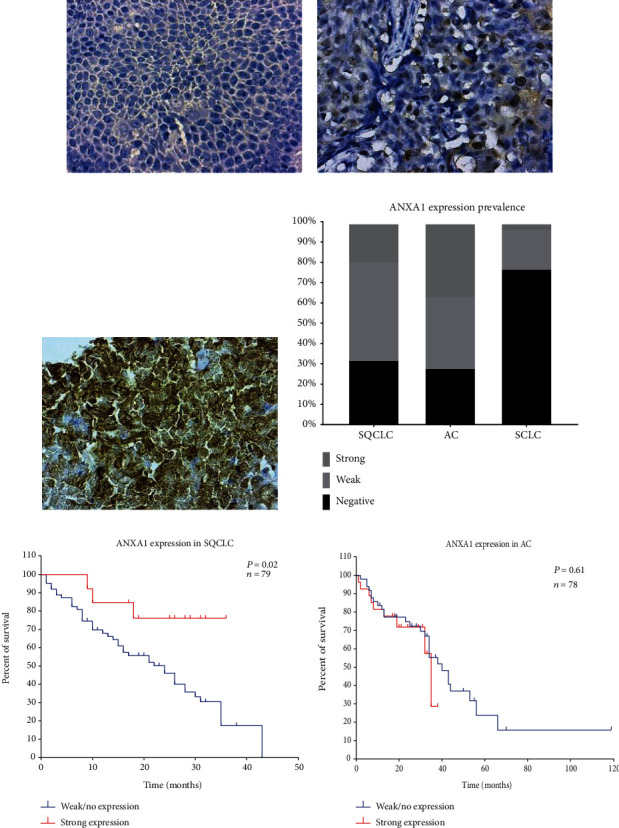
Immunohistochemistry staining of ANXA1 in lung cancer tissue samples. (a) Negative, (b) weak, and (c) strong staining of ANXA1 in SQCLC tissue samples. All images were captured at 400× magnification. (d) Prevalence of ANXA1 staining intensities in SQCLC, AC, and SCLC samples. (e and f) Kaplan-Meier curves correlating ANXA1 expression in SQCLC and AC tissue samples to patients' overall survival.

Tissue samples were stained for ANXA1 expression by immunohistochemistry (IHC). Strong ANXA1 expression was detected in 18.5% of SQCLC (15 of 81 samples), 36% of AC (31 of 86 samples), and 3.3% of SCLC (1 of 30 samples) (Figures 1(a)–1(d)).

Kaplan-Meier curves revealed a significant correlation between strong ANXA1 expression and increased overall survival of SQCLC patients (*P* = 0.019, HR: 3.5 and 95% CI: 1.7-7.3). The median survival of SQCLC patients with strong ANXA1 expression was 26 months, which was 9 months longer than negative/weak ANXA1 expression (17 months) (Figure 1(e)). Comparing ANXA1 expression to SQCLC patient's histopathological characteristics showed no correlation to sex, age, or patients' tumor stage (Table 2).

In the AC group, no correlation was found between ANXA1 expression and patient's overall survival (*P* = 0.613, HR: 0.8 and 95% CI: 0.4-1.8) while the limited number of strong expressing samples in the SCLC group (one) did not allow for survival analysis (Figure 1(f) and Supplementary Tables 2 and 3).

### 3.2. ANXA1 Promotes Proliferation of Human SQCLC Cell Lines

**Figure 2:**
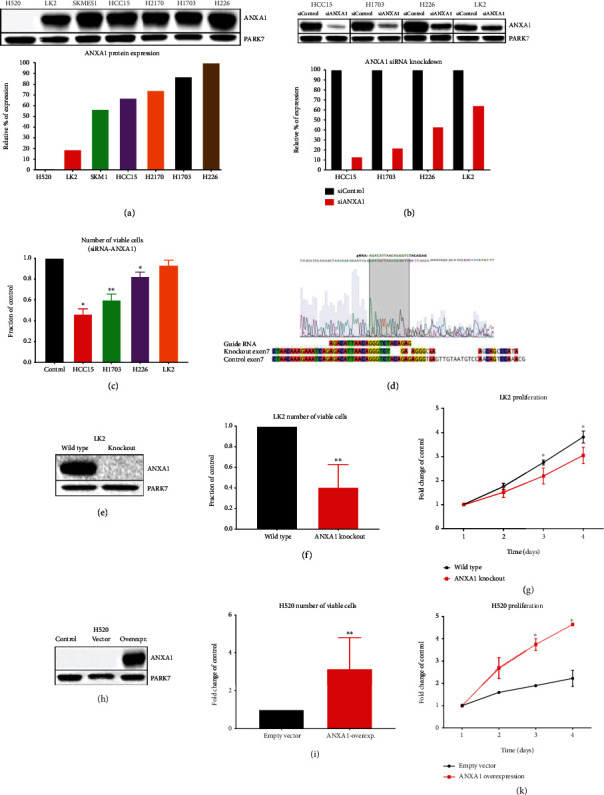
Correlation between ANXA1 expression and proliferation in SQCLC cell lines. (a) Western blot showing expression of ANXA1 in seven SQCLC cell lines. (b) Expression of ANXA1 in control-siRNA and ANXA1-siRNA treated cell lines. (c) Number of viable cells in ANXA1 compared to control siRNAs -treated cells after 120 hours. (d) Sanger sequencing showing exon seven knockout of ANXA1 gene in the LK2 cell line. (e) Western blot showing expression of ANXA1 protein in wild type and ANXA1 knockout of the LK2 cell line. (f and g) Number of viable cells (48 hours) and cellular proliferation in wild type compared to ANXA1 knockout LK2 cell line, respectively. (h) Overexpression of ANXA1 in the H520 cell line using a mammalian expression vector. (i and k) Number of viable cells (48 hours) and cellular proliferation in empty vector compared to ANXA1 transfected H520 cell lines. All experiments were held with three independent replicates.

Weakening of ANXA1 expression has shown to be significantly correlated to overall survival only in the SQCLC group; hence, we aimed at investigating the mechanistic role of ANXA1 in this group. In order to build a model for studying the functional role of ANXA1 in SQCLC cells, we analyzed the ANXA1 expression levels among seven SQCLC cell lines (H520, LK2, SKMES-1, HCC15, H2170, H1703, and H226) by western blotting. In each cell line, except of H520, specific bands at 37 kDa, the size of ANXA1, were detected. Accordingly, H520 seemed to be an ANXA1 low/nonexpressing cell line compared to the rest of human SQCLC cell lines (Figure 2(a)). PARK7 protein was used as a housekeeping protein due to its high expression stability among different cells and conditions [31]. Next, we knocked down ANXA1 expression using siRNA in the described SQCLC cell lines HCC15, H1703, H226, and LK2. Transfection efficiency of siRNA was verified through transfecting fluorescent-siRNA followed by analysis on FACS analyzer. The success of ANXA1 knockdown was evaluated through western blotting (Figure 2(b)). ANXA1 siRNA-knockdown significantly reduced the number of viable cells in HCC15 (45%), H1703 (59%), and H226 (82%) cell lines compared to the same cell lines transfected with control scrambled siRNA (Figure 2(c)). Reduction of viable cell number in LK2 cell line was the least (nonsignificant) compared to other cell lines, which could be reasoned back to the limited protein knockdown effect caused by siRNA compared to other cell lines (Figure 2(b)). Hence, we tested the effect of complete elimination (knockout) of ANXA1 expression using the CRISPR-Cas9 system in LK2 cell line on cell proliferation.

Disruption of the ANXA1 gene sequence was detected through DNA Sanger sequencing, and loss of protein expression was validated by western blotting (Figures 2(d) and 2(e)). The number of total viable cells of LK2-KO was significantly decreased compared to wild-type control (Figures 2(f) and 2(g)). Additionally, MTS proliferation assay was performed to compare the proliferation of LK2-KO to the LK2 wild type over a 4-day period. Comparing the two cell lines, a significant reduction in cell proliferation was detected in LK2-KO on days 3 and 4.

Wild type H520 cell line showed minimal/no expression of ANXA1 signals compared to other SQCLC cell lines. In order to test the effect of ANXA1 expression on proliferation in this cell line, we transfected it with either ANXA1 mammalian expressing vector or the same vector without the ANXA1 sequence. Western blot analysis confirmed overexpression of ANXA1 in the transfected cells (Figure 2(h)). Viable cell counting and MTS viability assay showed a significant increase of cell proliferation in ANXA1 overexpressing cells (Figures 2(i) and 2(k)).

### 3.3. ANXA1 Knockout Induces Migration of Human SQCLC Cell Lines

To investigate if ANXA1 protein expression plays a role in SQCLC cell metastasis, we performed migration assays using three different cell culture models. The first model included the LK2 cell line as control (LK2), ANXA1 knockout (LK2-KO), and ANXA1 knockout transfected with either empty vector (LK2-KO-Vector) or ANXA1 expression vector (LK2-KO-ANXA1). The second model included the H1703 cell line as control (H1703), ANXA1 knockout (H1703-KO), and ANXA1 knockout transfected with either empty vector (H1703-KO-Vector) or ANXA1 expression vector (H1703-KO-ANXA1). Guide RNAs had potential 0, 1, 1, and 10 off-target effects at 1, 2, 3, and 4 mismatches, respectively, with the full list mentioned in Supplementary Table 4. The last model included the H520 cell line as a control (H520) against H520 overexpressing ANXA1 (H520-ANXA1). Migration of tumor cells was measured until 120 hours in LK2 and H520 cell lines while only until 52 hours in H1703 cell line due to its relative original rapid growth rate.

**Figure 3:**
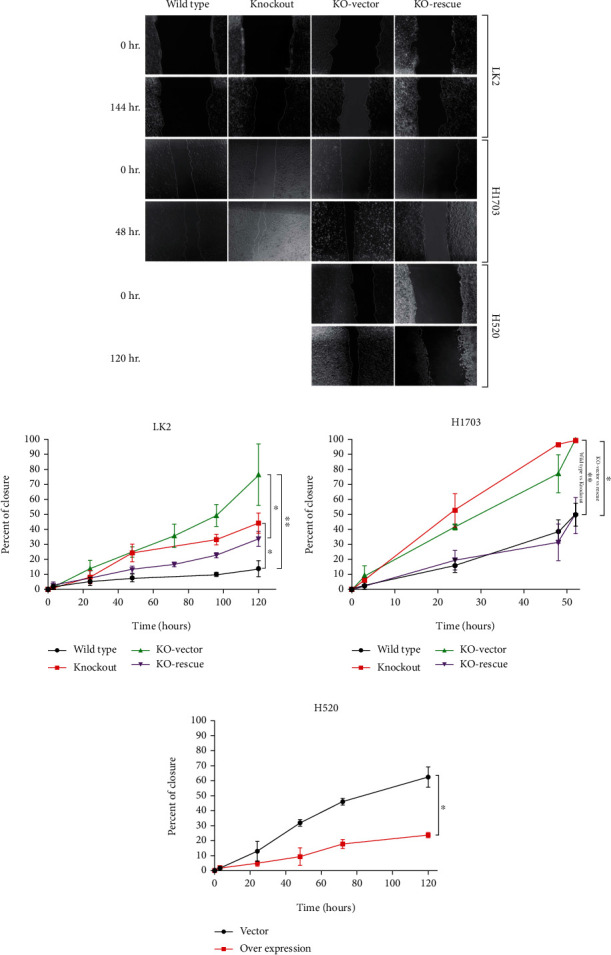
Effect of ANXA1 expression on cellular migration in SQCLC. (a) Representative microscopic images captured of LK2, Lk2-KO, LK2-ANXA1, and Lk2-empty vector at 0 and 48 hours; H1703, H1703-KO, H1703-empty, and H1703-ANXA1 at 0 and 144 hours; and H520-ANXA1 and H520-empty vector at 0 and 9 days from produced wounds. (b and c) Time needed for wound healing in ANXA1 wild type, knockout, and knockout rescued by overexpressing ANXA1 in LK2 and H1703 SQCLC cell lines. (f) Healing of scratch in H520 cells transfected with an empty vector compared to ANXA1 overexpressing vector. ^∗^ = *P* < 0.05, ^∗∗^ = *P* < 0.01, and ^∗∗∗^ = *P* < 0.001. Migration of cells was analyzed using ImageJ software. All experiments were held with three independent replicates.

The conducted migration assays indicated a significant reduction in time needed for wound healing in LK2-KO and H1703-KO compared to their wild-type controls. The elevated migration speed of ANXA1 knocked-out cell lines was rescued and reversed through transfection with ANXA1 expressing vectors (Figures 3(a)–3(c)). In the H520 cell line, overexpressing ANXA1 in H520-ANXA1 significantly elongated the migration time needed for cell healing (Figures 3(a) and 3(d)).

## 4. Discussion

Annexins are a family of Ca2+-regulated phospholipid-binding and membrane-binding proteins that play significant roles in cell cycle, exocytosis, and apoptosis [32, 33]. Different signaling pathways have been shown to be activated by ANXA1 including Src, PI3K/Akt, p38 MAPK, and MEK/ERK pathways [34, 35]. Meanwhile, in promoting cancer phenotypes, ANXA1 performs versatile functions [36, 37]. It preserves the malignant phenotype in pancreatic cancer cell lines, showing its potential to influence migration and invasion [35]. In breast cancer, ANXA1 overexpression is associated with resistance against 5-FU and trastuzumab treatments [38]. Additionally, ANXA1 expression was linked to reduced prognosis in gastric, bladder, breast, and renal cancers [14, 39–41].

The present study revealed that strong ANXA1 protein expression is significantly correlated to longer overall survival of patients with squamous cell lung cancer (SQCLC, *n* = 79) but not pulmonary adenocarcinoma (AC, *n* = 78). Even though several scholars have identified a significant increase of ANXA1 expression in different types of lung cancer, few have analyzed its impact on patients' survival [42, 43]. To the best of our knowledge, this study is the first to demonstrate that ANXA1 was a favorable prognostic factor for longer overall survival of SQCLC patients specifically. In other tumors, e.g., breast and epithelial ovarian cancer, clinical studies have observed a similar link between ANXA1 overexpression and longer overall survival, which was correlated to inhibition of tumor cells metastasis [44, 45]. Interestingly, ANXA1 expression did not correlate to any of patients' clinical features, e.g., age group, sex, lymph node metastasis, or degree of differentiation, which makes ANXA1 rather an independent prognostic marker than a specific marker to a certain group of patients.

In order to investigate the mechanism by which ANXA1 could have an effect on SQCLC patients' overall survival, we tested the impact of ANXA1 expression on proliferation and migration using multiple SQCLC cell lines. We did not test the effect of ANXA1 expression on cell lines of AC type due to lack of significant correlation in primary patient samples; nevertheless, the effect was previously studied *in vitro* [26]. Mutually transient and stable loss of ANXA1 expression led to proliferation inhibition in various SQCLC cell lines compared to controls, while overexpression of ANXA1 promoted SQCLC cell lines proliferation. However, we found an inverse correlation between the effect of ANXA1 expression on SQCLC cell proliferation and migration. Knocking out the ANXA1 gene using CRISPR-Cas9 significantly promoted cell migration in SQCLC cell lines, which was reversed and rescued through overexpressing the ANXA1 protein.

In our study, we considered that cell proliferation was not a relevant mechanism that interfered with the results of our migration assays, because rapidly proliferating cells migrated even more slowly than slow proliferating cell lines and vice versa.

Relating the results of our *in vitro* experiments to the patients' tissue samples data, we suggest that ANXA1 promotes SQCLC patients' overall survival through suppressing metastasis but not proliferation. This conclusion could be explained and supported by similar findings in breast cancer where migration properties rather than proliferation properties of cancer cells have shown more impact on tumor aggressiveness and patients overall survival [46]. Nonetheless, further research is necessary for clearer identification of the molecular mechanisms by which ANXA1 suppresses SQCLC migration.

In conclusion, our study showed a strong correlation between ANXA1 expression and longer overall survival of SQCLC patients. At the same time, the study provided evidence that ANXA1 promoted SQCLC cell lines proliferation but suppressed their migration *in vitro*. Our results highlighted ANXA1 as a potential prognostic marker for SQCLC that can predict prognosis and progression.

## Figures and Tables

**Table 1:** Patient characteristics.

Feature	Cases	SQCLC	AC	SCLC
Total	197	81	86	30
Age median (range)	67 (37-84)	67 (49-83)	68 (34-84)	67 (54-81)
Gender				
Male	132 (67%)	63 (47.7%)	47 (35.6%)	22 (16.7%)
Female	65 (33%)	18 (27.7%)	39 (60.0%)	8 (12.3%)
Age				
≥60	144 (73%)	59 (41%)	63 (43.8%)	22 (15.3%)
<60	53 (27%)	22 (41.5%)	23 (43.4%)	8 (15.1%)
Lymph node metastasis				
Yes	77 (41%)	37 (48.1%)	33 (42.9%)	7 (9.1%)
No	112 (59%)	44 (39.3%)	52 (46.4%)	16 (14.3%)
Degree of differentiation				
I+II	125 (63%)	64 (51.2%)	61 (48.8%)	0 (0.0%)
III	72 (37%)	17 (23.6%)	25 (34.7%)	30 (41.7%)
Clinical stage				
I+II	139 (74%)	56 (40.3%)	53 (45.3%)	20 (14.4%)
III+IV	50 (26%)	24 (48.0%)	23 (46.0%)	3 (6.0%)

Abbreviation: SQCLC: squamous cell lung cancer; AC: adenocarcinoma; SCLC: small cell.

**Table 2:** Immunohistochemistry analysis of SQCLC patient samples.

Features	Cases	ANXA1		*P* value
		—	+	
Gender				
Male	63	50 (79.4%)	13 (20.6%)	0.359
Female	18	16 (88.9%)	2 (11.1%)	
Age				
≥60	59	50 (84.7%)	9 (15.3%)	0.2155
<60	22	16 (72.7%)	6 (27.3%)	
Lymph node metastasis				
Yes	37	30 (81.1%)	7 (18.9%)	0.9322
No	44	36 (81.8%)	8 (18.2%)	
Degree of differentiation				
I+II	64	52 (81.3%)	12 (18.8%)	0.9171
III	17	14 (82.4%)	3 (17.6%)	
Clinical stage				
I+II	56	46 (82.1%)	10 (17.9%)	0.8978
III+IV	24	20 (83.3%)	4 (16.7%)	

SQCLC: squamous cell lung cancer, *P* values are calculated according to Chi-square test.

## Data Availability

All data that support the findings of the current study are available on request from the corresponding author. The data are not publicly available due to privacy or ethical restrictions.

## Supplementary Materials

Supplementary materials Supplementary Table 1: description of patient characteristics. Supplementary Table 2: analysis of immunohistochemistry staining of adenocarcinoma patient samples. Supplementary Table 3: analysis of immunohistochemistry staining of small cell lung cancer patient samples. Supplementary Table 4: list of off-targets score of ANXA1 knockout through CRISPR-Cas9.
