# Supplementary material for: Role of Annexin A1 in Squamous Cell Lung Cancer Progression
Source: Dis Markers. 2021 Apr 17;2021:5520832. doi: 10.1155/2021/5520832 (PMC8075699; doi:10.1155/2021/5520832)
Supplement: Supplementary materials — Supplementary Table 1: description of patient characteristics. Supplementary Table 2: analysis of immunohistochemistry staining of adenocarcinoma patient samples. Supplementary Table 3: analysis of immunohistochemistry staining of small cell lung cancer patient samples. Supplementary Table 4: list of off-targets score of ANXA1 knockout through CRISPR-Cas9. [file 5520832.f1.docx]

| **Supplementary table 1:**  **Patient characteristics** | | | | | | | | | | | |
| --- | --- | --- | --- | --- | --- | --- | --- | --- | --- | --- | --- |
| **#** | **Sex** | **Age** | **Histology** | **G** | **pT** | **pN** | **R** | **UICC** | **Overall survival [months]** | **OS-Status** | **IHC ANXA1** |
| 1 | male | 73 | Adenocarcinoma | 2 | 2 | 1 | 0 | 2 | 43 | deceased | negative |
| 2 | female | 68 | Adenocarcinoma | 3 | 2 | 1 | 0 | 2 | 23 | deceased | weak staining |
| 3 | female | 61 | Adenocarcinoma | 2 | 2 | 1 | 1 | 2 | 19 | deceased | strong staining |
| 4 | female | 72 | Adenocarcinoma | 3 | 4 | 0 | x | 4 | 6 | deceased | weak staining |
| 5 | female | 52 | Adenocarcinoma | 3 | 4 | 2 | 1 | 4 | 7 | deceased | weak staining |
| 6 | female | 49 | Adenocarcinoma | 3 | 2 | 0 | 0 | 4 | x | x | strong staining |
| 7 | male | 58 | Adenocarcinoma | 2 | 1 | x | 0 | 4 | 31 | alive | strong staining |
| 8 | male | 58 | Adenocarcinoma | 2 | 1 | 0 | 0 | 1 | 40 | deceased | negative |
| 9 | female | 49 | Adenocarcinoma | 2 | 1 | 0 | 0 | 1 | x | alive | negative |
| 10 | male | 58 | Adenocarcinoma | 2 | 1 | 0 | 0 | 1 | 37 | alive | negative |
| 11 | male | 75 | Adenocarcinoma | 2 | 1 | 0 | 0 | 1 | 12 | alive | negative |
| 12 | male | 63 | Adenocarcinoma | 2 | 1 | 0 | 0 | 1 | 11 | alive | negative |
| 13 | female | 67 | Adenocarcinoma | 2 | 1 | 0 | 0 | 1 | 50 | alive | negative |
| 14 | male | 58 | Adenocarcinoma | 3 | 1 | 0 | 0 | 1 | 1 | deceased | negative |
| 15 | female | 71 | Adenocarcinoma | 2 | 1 | 0 | 0 | 1 | 32 | deceased | weak staining |
| 16 | female | 39 | Adenocarcinoma | 2 | 1 | 0 | 0 | 1 | 53 | deceased | weak staining |
| 17 | female | 61 | Adenocarcinoma | 2 | 1 | 0 | 0 | 1 | 37 | alive | weak staining |
| 18 | female | 74 | Adenocarcinoma | 2 | 1 | 0 | x | 1 | 70 | alive | weak staining |
| 19 | female | 61 | Adenocarcinoma | 2 | 1 | 0 | 0 | 1 | 37 | alive | weak staining |
| 20 | male | 60 | Adenocarcinoma | 2 | 1 | 0 | 0 | 1 | 28 | alive | weak staining |
| 21 | female | 51 | Adenocarcinoma | 2 | 1 | 0 | 0 | 1 | 38 | alive | weak staining |
| 22 | male | 80 | Adenocarcinoma | 2 | 1 | 0 | 0 | 1 | 18 | alive | weak staining |
| 23 | female | 60 | Adenocarcinoma | 2 | 1 | 0 | 0 | 1 | 50 | alive | weak staining |
| 24 | female | 57 | Adenocarcinoma | 2 | 1 | 0 | 0 | 1 | 55 | alive | weak staining |
| 25 | male | 75 | Adenocarcinoma | 3 | 1 | 0 | 0 | 1 | 26 | alive | weak staining |
| 26 | female | 70 | Adenocarcinoma | 2 | 1 | 0 | 0 | 1 | 30 | deceased | weak staining |
| 27 | male | 51 | Adenocarcinoma | 2 | 1 | 0 | 0 | 1 | 44 | deceased | weak staining |
| 28 | female | 75 | Adenocarcinoma | 2 | 1 | 0 | 0 | 1 | 17 | alive | strong staining |
| 29 | female | 73 | Adenocarcinoma | 2 | 1 | 0 | 0 | 1 | x | x | strong staining |
| 30 | male | 71 | Adenocarcinoma | 3 | 1 | 0 | 0 | 1 | 18 | alive | strong staining |
| 31 | male | 63 | Adenocarcinoma | 2 | 1 | 0 | 0 | 1 | 20 | alive | strong staining |
| 32 | male | 57 | Adenocarcinoma | 3 | 1 | 0 | 0 | 1 | 21 | alive | strong staining |
| 33 | male | 73 | Adenocarcinoma | 2 | 1 | 0 | 0 | 1 | 34 | alive | strong staining |
| 34 | female | 70 | Adenocarcinoma | 2 | 1 | 0 | 0 | 1 | 17 | alive | strong staining |
| 35 | male | 80 | Adenocarcinoma | 2 | 1 | 0 | 0 | 1 | 18 | alive | strong staining |
| 36 | male | 74 | Adenocarcinoma | 2 | 1 | 0 | 0 | 1 | 29 | alive | strong staining |
| 37 | male | 73 | Adenocarcinoma | 2 | 2 | 0 | 0 | 1 | 25 | deceased | negative |
| 38 | male | 69 | Adenocarcinoma | 2 | 2 | 0 | 0 | 1 | 13 | deceased | negative |
| 39 | male | 60 | Adenocarcinoma | 2 | 2 | 0 | x | 1 | 43 | deceased | negative |
| 40 | male | 70 | Adenocarcinoma | 2 | 2 | 0 | 0 | 1 | 56 | deceased | weak staining |
| 41 | male | 76 | Adenocarcinoma | 2 | 2 | 0 | 0 | 1 | x | x | weak staining |
| 42 | female | 81 | Adenocarcinoma | 2 | 2 | 0 | 0 | 1 | 53 | alive | weak staining |
| 43 | male | 83 | Adenocarcinoma | 3 | 2 | 0 | x | 1 | 5 | deceased | weak staining |
| 44 | male | 60 | Adenocarcinoma | 2 | 2 | 0 | 0 | 1 | 38 | alive | strong staining |
| 45 | female | 65 | Adenocarcinoma | 2 | 2 | 0 | 0 | 1 | 20 | alive | strong staining |
| 46 | female | 81 | Adenocarcinoma | 3 | 2 | 0 | 0 | 1 | 18 | alive | strong staining |
| 47 | male | 73 | Adenocarcinoma | 3 | 1 | 1 | 0 | 2 | 18 | alive | negative |
| 48 | female | 68 | Adenocarcinoma | 2 | 2 | 0 | 0 | 2 | 44 | alive | weak staining |
| 49 | female | 69 | Adenocarcinoma | 2 | 2 | 0 | 1 | 2 | 119 | alive | weak staining |
| 50 | male | 64 | Adenocarcinoma | 3 | 1 | 1 | 0 | 2 | 8 | deceased | weak staining |
| 51 | male | 70 | Adenocarcinoma | 2 | 2 | 1 | 0 | 2 | 7 | deceased | weak staining |
| 52 | male | 61 | Adenocarcinoma | 2 | 2 | 1 | 0 | 2 | 14 | x | weak staining |
| 53 | female | 59 | Adenocarcinoma | 2 | 1 | 1 | 0 | 2 | 17 | alive | strong staining |
| 54 | female | 74 | Adenocarcinoma | 2 | 1 | 1 | 0 | 2 | 6 | deceased | strong staining |
| 55 | male | 54 | Adenocarcinoma | 3 | 2 | 1 | 0 | 2 | 7 | deceased | strong staining |
| 56 | male | 70 | Adenocarcinoma | 2 | 3 | 0 | 0 | 2 | 19 | alive | negative |
| 57 | female | 34 | Adenocarcinoma | 2 | 3 | 0 | 0 | 2 | 18 | alive | negative |
| 58 | female | 59 | Adenocarcinoma | 2 | 3 | 0 | 0 | 2 | 32 | alive | negative |
| 59 | male | 55 | Adenocarcinoma | 3 | 3 | 0 | 0 | 2 | 13 | deceased | negative |
| 60 | female | 59 | Adenocarcinoma | 2 | 2 | 2 | 0 | 2 | 66 | deceased | negative |
| 61 | male | 70 | Adenocarcinoma | 2 | 3 | 0 | 0 | 2 | 19 | alive | weak staining |
| 62 | female | 53 | Adenocarcinoma | 2 | 3 | 0 | 0 | 2 | 32 | deceased | strong staining |
| 63 | male | 77 | Adenocarcinoma | 2 | 3 | 0 | 0 | 2 | 1 | deceased | strong staining |
| 64 | male | 72 | Adenocarcinoma | 3 | 3 | 0 | 0 | 2 | 8 | deceased | strong staining |
| 65 | male | 57 | Adenocarcinoma | 3 | 3 | 0 | 0 | 2 | 18 | alive | strong staining |
| 66 | male | 69 | Adenocarcinoma | 2 | 2 | 1 | 0 | 2 | 31 | alive | strong staining |
| 67 | male | 64 | Adenocarcinoma | 2 | 2 | 1 | 0 | 2 | 33 | alive | strong staining |
| 68 | male | 84 | Adenocarcinoma | 3 | 3 | 1 | 0 | 3 | 2 | deceased | negative |
| 69 | female | 69 | Adenocarcinoma | 3 | 4 | 1 | 0 | 3 | 33 | alive | negative |
| 70 | male | 69 | Adenocarcinoma | 3 | 1 | 2 | 0 | 3 | 34 | deceased | negative |
| 71 | female | 80 | Adenocarcinoma | 2 | 2 | 2 | 0 | 3 | 5 | deceased | negative |
| 72 | female | 78 | Adenocarcinoma | 3 | 2 | 2 | 0 | 3 | x | deceased | negative |
| 73 | male | 77 | Adenocarcinoma | 2 | 2 | 2 | 0 | 3 | 12 | deceased | negative |
| 74 | male | 69 | Adenocarcinoma | 3 | 1 | 2 | 0 | 3 | 34 | deceased | weak staining |
| 75 | male | 63 | Adenocarcinoma | 2 | 2 | 2 | 0 | 3 | 34 | deceased | weak staining |
| 76 | male | 63 | Adenocarcinoma | 2 | 2 | 2 | 0 | 3 | 34 | deceased | weak staining |
| 77 | female | 44 | Adenocarcinoma | 2 | 4 | 2 | 1 | 3 | 38 | deceased | weak staining |
| 78 | female | 76 | Adenocarcinoma | 2 | 1 | 2 | 0 | 3 | x | x | weak staining |
| 79 | female | 67 | Adenocarcinoma | 3 | 3 | 1 | 0 | 3 | 2 | deceased | strong staining |
| 80 | female | 58 | Adenocarcinoma | 3 | 3 | 2 | 0 | 3 | 17 | alive | strong staining |
| 81 | female | 76 | Adenocarcinoma | 2 | 1 | 2 | 0 | 3 | x | x | strong staining |
| 82 | male | 72 | Adenocarcinoma | 2 | 1 | 2 | 0 | 3 | 35 | deceased | strong staining |
| 83 | male | 70 | Adenocarcinoma | 2 | 2 | 2 | 0 | 3 | 24 | alive | strong staining |
| 84 | male | 62 | Adenocarcinoma | 3 | 2 | 2 | 0 | 3 | x | x | strong staining |
| 85 | male | 62 | Adenocarcinoma | 3 | 2 | 2 | 0 | 3 | 13 | deceased | strong staining |
| 86 | female | 53 | Adenocarcinoma | 2 | 4 | 2 | 0 | 3 | 10 | deceased | negative |
| 87 | female | 79 | Squamous cell carcinoma | 2 | 1 | 0 | 0 | 1 | 10 | alive | negative |
| 88 | male | 75 | Squamous cell carcinoma | 2 | 1 | 0 | 0 | 1 | x | x | negative |
| 89 | male | 67 | Squamous cell carcinoma | 2 | 1 | 0 | 0 | 1 | 28 | alive | negative |
| 90 | male | 73 | Squamous cell carcinoma | 2 | 1 | 0 | 0 | 1 | 21 | deceased | negative |
| 91 | male | 58 | Squamous cell carcinoma | 2 | 1 | 0 | 0 | 1 | 30 | alive | weak staining |
| 92 | female | 75 | Squamous cell carcinoma | 3 | 1 | 0 | 0 | 1 | 7 | deceased | weak staining |
| 93 | male | 63 | Squamous cell carcinoma | 3 | 1 | 0 | 0 | 1 | 31 | deceased | weak staining |
| 94 | male | 57 | Squamous cell carcinoma | 2 | 1 | 0 | 0 | 1 | 19 | alive | weak staining |
| 95 | male | 56 | Squamous cell carcinoma | 2 | 1 | 0 | 1 | 1 | 32 | alive | weak staining |
| 96 | female | 70 | Squamous cell carcinoma | 2 | 1 | 0 | 0 | 1 | 28 | alive | weak staining |
| 97 | female | 70 | Squamous cell carcinoma | 2 | 1 | 0 | 0 | 1 | 26 | alive | weak staining |
| 98 | female | 80 | Squamous cell carcinoma | 2 | 1 | 0 | 0 | 1 | 8 | deceased | weak staining |
| 99 | male | 76 | Squamous cell carcinoma | 2 | 1 | 0 | 0 | 1 | 22 | deceased | weak staining |
| 100 | female | 62 | Squamous cell carcinoma | 2 | 1 | 0 | 0 | 1 | 8 | deceased | weak staining |
| 101 | male | 76 | Squamous cell carcinoma | 2 | 1 | 0 | 0 | 1 | x | x | strong staining |
| 102 | female | 72 | Squamous cell carcinoma | 2 | 1 | 0 | 0 | 1 | 19 | alive | strong staining |
| 103 | male | 58 | Squamous cell carcinoma | 2 | 1 | 0 | 0 | 1 | 31 | alive | strong staining |
| 104 | male | 59 | Squamous cell carcinoma | 3 | 1 | 0 | 0 | 1 | 26 | alive | strong staining |
| 105 | male | 82 | Squamous cell carcinoma | 2 | 2 | 0 | 1 | 1 | 6 | deceased | negative |
| 106 | male | 62 | Squamous cell carcinoma | 3 | 2 | 0 | 0 | 1 | 11 | alive | negative |
| 107 | female | 65 | Squamous cell carcinoma | 2 | 2 | 0 | 0 | 1 | 26 | deceased | weak staining |
| 108 | male | 74 | Squamous cell carcinoma | 2 | 2 | 0 | 0 | 1 | 30 | deceased | weak staining |
| 109 | male | 72 | Squamous cell carcinoma | 2 | 2 | 0 | 0 | 1 | 1 | deceased | weak staining |
| 110 | male | 60 | Squamous cell carcinoma | 3 | 2 | 0 | 0 | 1 | 10 | deceased | weak staining |
| 111 | male | 60 | Squamous cell carcinoma | 2 | 2 | 0 | x | 1 | 43 | deceased | weak staining |
| 112 | male | 67 | Squamous cell carcinoma | 2 | 2 | 0 | 0 | 1 | 25 | alive | strong staining |
| 113 | male | 63 | Squamous cell carcinoma | 2 | 2 | 0 | 1 | 2 | 2 | deceased | negative |
| 114 | female | 63 | Squamous cell carcinoma | 2 | 2 | 0 | 0 | 2 | 32 | alive | negative |
| 115 | female | 69 | Squamous cell carcinoma | 2 | 2 | 0 | 0 | 2 | 26 | deceased | negative |
| 116 | male | 64 | Squamous cell carcinoma | 2 | 2 | 1 | 0 | 2 | 11 | alive | negative |
| 117 | female | 58 | Squamous cell carcinoma | 2 | 2 | 1 | 0 | 2 | 38 | alive | negative |
| 118 | male | 49 | Squamous cell carcinoma | 2 | 2 | 1 | 0 | 2 | 26 | deceased | negative |
| 119 | male | 65 | Squamous cell carcinoma | 2 | 2 | 1 | 0 | 2 | 8 | deceased | negative |
| 120 | female | 75 | Squamous cell carcinoma | 2 | 2 | 1 | 0 | 2 | 35 | deceased | negative |
| 121 | male | 69 | Squamous cell carcinoma | 3 | 2 | 1 | 0 | 2 | 35 | alive | negative |
| 122 | male | 71 | Squamous cell carcinoma | 2 | 2 | 0 | 0 | 2 | 24 | deceased | negative |
| 123 | male | 66 | Squamous cell carcinoma | 2 | 2 | 0 | 0 | 2 | 6 | deceased | weak staining |
| 124 | female | 59 | Squamous cell carcinoma | 2 | 2 | 1 | 1 | 2 | 24 | deceased | weak staining |
| 125 | male | 59 | Squamous cell carcinoma | 2 | 2 | 1 | 0 | 2 | 16 | deceased | weak staining |
| 126 | male | 56 | Squamous cell carcinoma | 2 | 2 | 1 | 1 | 2 | 28 | deceased | weak staining |
| 127 | male | 76 | Squamous cell carcinoma | 2 | 2 | 0 | 0 | 2 | 29 | alive | strong staining |
| 128 | male | 57 | Squamous cell carcinoma | 2 | 1 | 1 | 0 | 2 | 36 | alive | strong staining |
| 129 | male | 76 | Squamous cell carcinoma | 2 | 2 | 1 | 0 | 2 | 9 | deceased | strong staining |
| 130 | male | 53 | Squamous cell carcinoma | 2 | 2 | 1 | 0 | 2 | 10 | deceased | strong staining |
| 131 | male | 66 | Squamous cell carcinoma | 2 | 3 | 0 | 0 | 2 | 16 | deceased | negative |
| 132 | female | 67 | Squamous cell carcinoma | 2 | 3 | 0 | 0 | 2 | 38 | alive | negative |
| 133 | male | 73 | Squamous cell carcinoma | 3 | 3 | 0 | 0 | 2 | 8 | deceased | negative |
| 134 | male | 72 | Squamous cell carcinoma | 3 | 3 | 0 | 0 | 2 | 1 | deceased | negative |
| 135 | female | 70 | Squamous cell carcinoma | 2 | 3 | 0 | 0 | 2 | 10 | deceased | weak staining |
| 136 | male | 69 | Squamous cell carcinoma | 2 | 3 | 0 | 0 | 2 | 20 | alive | weak staining |
| 137 | male | 75 | Squamous cell carcinoma | 2 | 3 | 0 | 0 | 2 | 15 | deceased | weak staining |
| 138 | male | 49 | Squamous cell carcinoma | 2 | 3 | 0 | 0 | 2 | 1 | deceased | weak staining |
| 139 | male | 65 | Squamous cell carcinoma | 2 | 3 | 0 | 0 | 2 | 35 | deceased | weak staining |
| 140 | male | 68 | Squamous cell carcinoma | 2 | 3 | 0 | 0 | 2 | 35 | deceased | weak staining |
| 141 | male | 67 | Squamous cell carcinoma | 2 | 2 | 1 | 0 | 2 | 28 | deceased | weak staining |
| 142 | male | 76 | Squamous cell carcinoma | 2 | 3 | 0 | 0 | 2 | x | x | strong staining |
| 143 | male | 54 | Squamous cell carcinoma | 2 | 3 | 1 | 0 | 3 | 12 | deceased | negative |
| 144 | male | 55 | Squamous cell carcinoma | 2 | 3 | 1 | 0 | 3 | 1 | deceased | negative |
| 145 | male | 73 | Squamous cell carcinoma | 2 | 3 | 1 | 0 | 3 | 28 | alive | negative |
| 146 | male | 55 | Squamous cell carcinoma | 3 | 4 | 1 | 0 | 3 | 15 | deceased | negative |
| 147 | female | 60 | Squamous cell carcinoma | 2 | 2 | 2 | 0 | 3 | 32 | alive | negative |
| 148 | male | 81 | Squamous cell carcinoma | 2 | 3 | 1 | 0 | 3 | 4 | deceased | weak staining |
| 149 | male | 57 | Squamous cell carcinoma | 2 | 3 | 1 | 0 | 3 | 1 | deceased | weak staining |
| 150 | male | 60 | Squamous cell carcinoma | 3 | 3 | 1 | 0 | 3 | 23 | alive | weak staining |
| 151 | male | 69 | Squamous cell carcinoma | 3 | 3 | 1 | 0 | 3 | 6 | deceased | weak staining |
| 152 | male | 54 | Squamous cell carcinoma | 2 | 4 | 1 | 0 | 3 | 3 | deceased | weak staining |
| 153 | male | 71 | Squamous cell carcinoma | 2 | 4 | 1 | 1 | 3 | 31 | alive | weak staining |
| 154 | male | 83 | Squamous cell carcinoma | 3 | 4 | 1 | 0 | 3 | 10 | deceased | weak staining |
| 155 | male | 59 | Squamous cell carcinoma | 3 | 4 | 1 | 0 | 3 | 13 | deceased | weak staining |
| 156 | male | 67 | Squamous cell carcinoma | 2 | 1 | 1 | 0 | 3 | 17 | deceased | weak staining |
| 157 | male | 66 | Squamous cell carcinoma | 3 | 3 | 2 | 0 | 3 | 3 | deceased | weak staining |
| 158 | male | 76 | Squamous cell carcinoma | 2 | 1 | 2 | 0 | 3 | 14 | deceased | weak staining |
| 159 | male | 67 | Squamous cell carcinoma | 2 | 2 | 2 | 0 | 3 | 21 | deceased | weak staining |
| 160 | female | 71 | Squamous cell carcinoma | 2 | 2 | 2 | 0 | 3 | 21 | alive | weak staining |
| 161 | male | 78 | Squamous cell carcinoma | 2 | 4 | 0 | 0 | 3 | 18 | deceased | strong staining |
| 162 | male | 54 | Squamous cell carcinoma | 3 | 3 | 1 | 0 | 3 | 32 | alive | strong staining |
| 163 | female | 56 | Squamous cell carcinoma | 3 | 3 | 2 | 1 | 3 | 28 | alive | strong staining |
| 164 | male | 60 | Squamous cell carcinoma | 2 | 2 | 2 | 0 | 3 | 28 | alive | strong staining |
| 165 | male | 53 | Squamous cell carcinoma | 2 | 4 | 2 | 0 | 3 | 2 | deceased | negative |
| 166 | male | 75 | Squamous cell carcinoma | 3 | 4 | 2 | 0 | 3 | 9 | alive | weak staining |
| 167 | male | 75 | Squamous cell carcinoma | 2 | x | 1 | 1 | x | 17 | alive | strong staining |
| 168 | male | 70 | Small cell lung cancer | 3 | 1 | 0 | 0 | 1 | 7 | deceased | negative |
| 169 | male | 56 | Small cell lung cancer | 3 | 1 | 0 | 0 | 1 | 1 | deceased | negative |
| 170 | male | 67 | Small cell lung cancer | 3 | 1 | 0 | 0 | 1 | 28 | deceased | negative |
| 171 | male | 77 | Small cell lung cancer | 3 | 1 | 0 | 0 | 1 | 72 | alive | negative |
| 172 | male | 67 | Small cell lung cancer | 3 | 1 | 0 | 0 | 1 | 13 | deceased | negative |
| 173 | male | 72 | Small cell lung cancer | 3 | 1 | 0 | 0 | 1 | 42 | alive | negative |
| 174 | female | 81 | Small cell lung cancer | 3 | 1 | 0 | 0 | 1 | x | x | negative |
| 175 | male | 60 | Small cell lung cancer | 3 | 1 | 0 | 0 | 1 | 12 | deceased | negative |
| 176 | male | 71 | Small cell lung cancer | 3 | 1 | 0 | 0 | 1 | 51 | alive | negative |
| 177 | male | 76 | Small cell lung cancer | 3 | 1 | 0 | 0 | 1 | 18 | deceased | negative |
| 178 | male | 65 | Small cell lung cancer | 3 | 1 | 0 | 0 | 1 | 106 | alive | weak staining |
| 179 | male | 73 | Small cell lung cancer | 3 | 1 | 0 | 0 | 1 | 57 | alive | weak staining |
| 180 | female | 58 | Small cell lung cancer | 3 | 1 | 0 | 0 | 1 | 9 | deceased | weak staining |
| 181 | male | 56 | Small cell lung cancer | 3 | 2 | 0 | 0 | 1 | 76 | alive | negative |
| 182 | male | 54 | Small cell lung cancer | 3 | 2 | 0 | 0 | 1 | 52 | alive | negative |
| 183 | female | 59 | Small cell lung cancer | 3 | 1 | 1 | 0 | 2 | 66 | deceased | negative |
| 184 | female | 60 | Small cell lung cancer | 3 | 2 | 1 | x | 2 | 125 | alive | negative |
| 185 | male | 75 | Small cell lung cancer | 3 | 2 | 1 | 0 | 2 | x | x | weak staining |
| 186 | female | 59 | Small cell lung cancer | 3 | 1 | 1 | 0 | 2 | x | alive | strong staining |
| 187 | male | 68 | Small cell lung cancer | 3 | 3 | 0 | 0 | 2 | 9 | deceased | negative |
| 188 | male | 74 | Small cell lung cancer | 3 | 1 | 2 | 1 | 3 | 2 | deceased | negative |
| 189 | female | 56 | Small cell lung cancer | 3 | 1 | 2 | 1 | 3 | 23 | deceased | negative |
| 190 | male | 56 | Small cell lung cancer | 3 | 1 | 2 | 0 | 3 | 9 | deceased | weak staining |
| 191 | male | 69 | Small cell lung cancer | 3 | 1 | x | x | x | 1 | deceased | negative |
| 192 | male | 63 | Small cell lung cancer | 3 | 3 | x | 0 | x | 25 | deceased | negative |
| 193 | male | 68 | Small cell lung cancer | 3 | 1 | x | 1 | x | 51 | alive | negative |
| 194 | female | 65 | Small cell lung cancer | 3 | 1 | x | 0 | x | 31 | alive | negative |
| 195 | male | 63 | Small cell lung cancer | 3 | 1 | x | 0 | x | 34 | deceased | negative |
| 196 | female | 72 | Small cell lung cancer | 3 | x | x | 1 | x | 5 | deceased | negative |
| 197 | male | 73 | Small cell lung cancer | 3 | 1 | x | x | x | 29 | deceased | weak staining |

| \| **Supplementary table 2:**  **Immunohistochemistry analysis of AC patient samples** \| \| \| \| \| \| --- \| --- \| --- \| --- \| --- \| \|  \| ANXA1 \| \| \| \| \| Features \| Cases \| - \| + \| P value \| \| Gender \|  \|  \|  \|  \| \| Male \| 47 \| 29 (61.7%) \| 18 (38.3%) \| 0.6331 \| \| Female \| 39 \| 26 (66.7%) \| 13 (33.3%) \| \| Age \|  \|  \|  \|  \| \| ≥60 \| 63 \| 40 (63.5%) \| 23 (36.5%) \| 0.8827 \| \| <60 \| 23 \| 15 (65.2%) \| 8 (34.8%) \| \| Lymph node metastasis \|  \|  \|  \|  \| \| Yes \| 33 \| 20 (60.6%) \| 13 (39.4%) \| 0.5286 \| \| No \| 52 \| 35 (67.3%) \| 17 (32.7%) \| \| Degree of differentiation \|  \|  \|  \|  \| \| I+II \| 61 \| 41 (67.2%) \| 20 (32.8%) \| 0.3254 \| \| III \| 25 \| 14 (56.0%) \| 11 (44.0%) \| \| Clinical stage \|  \|  \|  \|  \| \| I+II \| 63 \| 41 (65.1%) \| 22 (34.9%) \| 0.7189 \| \| III+IV \| 23 \| 14 (60.9%) \| 9 (39.1%) \| \| AC: Adenocarcinoma of the lung, p values are calculated according to Chi-Square test. \| \| \| \| \| | | | | |
| --- | --- | --- | --- | --- | --- | --- | --- | --- | --- | --- | --- | --- | --- | --- | --- | --- | --- | --- | --- | --- | --- | --- | --- | --- | --- | --- | --- | --- | --- | --- | --- | --- | --- | --- | --- | --- | --- | --- | --- | --- | --- | --- | --- | --- | --- | --- | --- | --- | --- | --- | --- | --- | --- | --- | --- | --- | --- | --- | --- | --- | --- | --- | --- | --- | --- | --- | --- | --- | --- | --- | --- | --- | --- | --- | --- | --- | --- | --- | --- | --- | --- | --- | --- | --- | --- | --- | --- | --- | --- | --- | --- | --- | --- | --- |
|  |  | | | |
|  |  |  |  |  |

| \| **Supplementary table 3:**  **Immunohistochemistry analysis of SCLC patient samples** \| \| \| \| \| \| --- \| --- \| --- \| --- \| --- \| \|  \| ANXA1 \| \| \| \| \| Features \| Cases \| - \| + \| P value \| \| Gender \|  \|  \|  \|  \| \| Male \| 22 \| 22 (100.0%) \| 0 (0.0%) \| 0.0917 \| \| Female \| 8 \| 7 (87.5%) \| 1 (12.5%) \| \| Age \|  \|  \|  \|  \| \| ≥60 \| 22 \| 22 (100.0%) \| 0 (0.0%) \| 0.0917 \| \| <60 \| 8 \| 7 (87.5%) \| 1 (12.5%) \| \| Lymph node metastasis \|  \|  \|  \|  \| \| Yes \| 7 \| 6 (85.7%) \| 1 (14.3%) \| 0.1221 \| \| No \| 16 \| 16 (100.0%) \| 0 (0.0%) \| \| Degree of differentiation \|  \|  \|  \|  \| \| I+II \| 0 \| 0 (0.0%) \| 0 (0.0%) \| - \| \| III \| 30 \| 29 (96.7%) \| 1 (3.3%) \| \| Clinical stage \|  \|  \|  \|  \| \| I+II \| 20 \| 19 (95.0%) \| 1 (5.0%) \| 0.6921 \| \| III+IV \| 3 \| 3 (100.0%) \| 0 (0.0%) \| \|  \|  \|  \|  \|  \| \| SCLC: Small cell lung cancer, p values are calculated according to Chi-Square test. \| \| \| \| \| |  |  |  |  |
| --- | --- | --- | --- | --- | --- | --- | --- | --- | --- | --- | --- | --- | --- | --- | --- | --- | --- | --- | --- | --- | --- | --- | --- | --- | --- | --- | --- | --- | --- | --- | --- | --- | --- | --- | --- | --- | --- | --- | --- | --- | --- | --- | --- | --- | --- | --- | --- | --- | --- | --- | --- | --- | --- | --- | --- | --- | --- | --- | --- | --- | --- | --- | --- | --- | --- | --- | --- | --- | --- | --- | --- | --- | --- | --- | --- | --- | --- | --- | --- | --- | --- | --- | --- | --- | --- | --- | --- | --- | --- | --- | --- | --- | --- | --- | --- | --- | --- | --- | --- |
|  |  |  |  |  |
|  |  |  |  |  |
|  |  |  |  |  |
|  |  |  |  |  |
|  |  |  |  |  |
|  |  |  |  |  |
|  |  |  |  |  |
|  |  |  |  |  |
|  |  |  |  |  |
|  |  |  |  |  |
|  |  |  |  |  |
| \| **Supplementary table 4:**  **Genome Browser links to matches sorted by CFD off-target score**  **ACCORDING TO CRISPOR.TEFOR.NET** \| \| \| --- \| --- \| \| gRNA: fwd: CACCGAGACATTAACAGGGTCTACAGAG, rev: AAACCTCTGTAGACCCTGTTAATGTCTC \| \| \| 1 \| [3:intergenic:DKK2-SNORD112](http://genome.ucsc.edu/cgi-bin/hgTracks?db=hg19&position=chr4:107985542-107985564) \| \| 2 \| [4:intergenic:RFX6-RPS29P13](http://genome.ucsc.edu/cgi-bin/hgTracks?db=hg19&position=chr6:117301615-117301637) \| \| 3 \| [4:intergenic:AF015720.3-RUNX1](http://genome.ucsc.edu/cgi-bin/hgTracks?db=hg19&position=chr21:37111435-37111457) \| \| 4 \| [4:intergenic:RP5-827L5.1-CDH4](http://genome.ucsc.edu/cgi-bin/hgTracks?db=hg19&position=chr20:59704207-59704229) \| \| 5 \| [4:intron:NCAM2](http://genome.ucsc.edu/cgi-bin/hgTracks?db=hg19&position=chr21:22406187-22406209) \| \| 6 \| [4:intron:IL1RAPL1](http://genome.ucsc.edu/cgi-bin/hgTracks?db=hg19&position=chrX:29813035-29813057) \| \| 7 \| [4:intergenic:CDC5L-RP3-449H6.1](http://genome.ucsc.edu/cgi-bin/hgTracks?db=hg19&position=chr6:44465900-44465922) \| \| 8 \| [4:intron:OFCC1](http://genome.ucsc.edu/cgi-bin/hgTracks?db=hg19&position=chr6:9988210-9988232) \| \| 9 \| [4:intergenic:RNU6-1271P-RP11-54A9.1](http://genome.ucsc.edu/cgi-bin/hgTracks?db=hg19&position=chr12:76576552-76576574) \| \| 10 \| [4:intergenic:ESX1-IL1RAPL2](http://genome.ucsc.edu/cgi-bin/hgTracks?db=hg19&position=chrX:103503538-103503560) \| \| 11 \| [3:intergenic:RP11-813P10.2-AC091517.1](http://genome.ucsc.edu/cgi-bin/hgTracks?db=hg19&position=chr12:60223955-60223977) \| \| 12 \| [4:intergenic:NAMA-RP11-547C13.1](http://genome.ucsc.edu/cgi-bin/hgTracks?db=hg19&position=chr9:102161358-102161380) \| \| 13 \| [4:intron:RNF185](http://genome.ucsc.edu/cgi-bin/hgTracks?db=hg19&position=chr22:31574259-31574281) \| \| 14 \| [4:exon:C12orf66](http://genome.ucsc.edu/cgi-bin/hgTracks?db=hg19&position=chr12:64581126-64581148) \| \| 15 \| [4:intron:AC007246.3](http://genome.ucsc.edu/cgi-bin/hgTracks?db=hg19&position=chr2:39736444-39736466) \| \| 16 \| [4:intergenic:KHDRBS1-RP11-277A4.4](http://genome.ucsc.edu/cgi-bin/hgTracks?db=hg19&position=chr1:32511207-32511229) \| \| 17 \| [4:intron:ANO2](http://genome.ucsc.edu/cgi-bin/hgTracks?db=hg19&position=chr12:5861052-5861074) \| \| 18 \| [4:intergenic:ABLIM3-RP11-331K21.1](http://genome.ucsc.edu/cgi-bin/hgTracks?db=hg19&position=chr5:148533118-148533140) \| \| 19 \| [3:intron:NMNAT3](http://genome.ucsc.edu/cgi-bin/hgTracks?db=hg19&position=chr3:139374006-139374028) \| \| 20 \| [4:intergenic:RP11-2L13.1-RP11-567B20.1](http://genome.ucsc.edu/cgi-bin/hgTracks?db=hg19&position=chr1:199779184-199779206) \| \| 21 \| [4:exon:FAM156A](http://genome.ucsc.edu/cgi-bin/hgTracks?db=hg19&position=chrX:52995040-52995062) \| \| 22 \| [4:intergenic:XAGE3-FAM156B](http://genome.ucsc.edu/cgi-bin/hgTracks?db=hg19&position=chrX:52918969-52918991) \| \| 23 \| [4:intergenic:RP11-524L6.4-RP11-524L6.3/BIN2P2](http://genome.ucsc.edu/cgi-bin/hgTracks?db=hg19&position=chr5:75187049-75187071) \| \| 24 \| [4:intergenic:NDFIP2/NDFIP2-AS1-NDFIP2](http://genome.ucsc.edu/cgi-bin/hgTracks?db=hg19&position=chr13:80064103-80064125) \| \| 25 \| [2:intergenic:RP11-616L12.1-NEUROD4](http://genome.ucsc.edu/cgi-bin/hgTracks?db=hg19&position=chr12:55410124-55410146) \| \| 26 \| [4:intron:HYDIN](http://genome.ucsc.edu/cgi-bin/hgTracks?db=hg19&position=chr16:71237989-71238011) \| \| 27 \| [4:intron:C2orf43](http://genome.ucsc.edu/cgi-bin/hgTracks?db=hg19&position=chr2:21006076-21006098) \| \| 28 \| [4:intergenic:RP11-10C8.2-MTND4P7](http://genome.ucsc.edu/cgi-bin/hgTracks?db=hg19&position=chr8:13210149-13210171) \| \| 29 \| [4:intergenic:SEMA6D-RP11-142J21.2](http://genome.ucsc.edu/cgi-bin/hgTracks?db=hg19&position=chr15:47797688-47797710) \| \| 30 \| [1:intron:EZR](http://genome.ucsc.edu/cgi-bin/hgTracks?db=hg19&position=chr6:159199569-159199591) \| \| 31 \| [4:intergenic:RP11-386M24.3-RP11-386M24.9](http://genome.ucsc.edu/cgi-bin/hgTracks?db=hg19&position=chr15:93146010-93146032) \| \| 32 \| [4:intergenic:OTUD1-KIAA1217](http://genome.ucsc.edu/cgi-bin/hgTracks?db=hg19&position=chr10:23773532-23773554) \| \| 33 \| [4:intron:SPIDR](http://genome.ucsc.edu/cgi-bin/hgTracks?db=hg19&position=chr8:48249819-48249841) \| \| 34 \| [4:exon:SH3PXD2A](http://genome.ucsc.edu/cgi-bin/hgTracks?db=hg19&position=chr10:105360834-105360856) \| \| 35 \| [4:intergenic:RP11-453O22.1-RP11-421L10.1](http://genome.ucsc.edu/cgi-bin/hgTracks?db=hg19&position=chr1:221351722-221351744) \| \| 36 \| [4:intergenic:RP11-424D14.1-RP11-74K19.1](http://genome.ucsc.edu/cgi-bin/hgTracks?db=hg19&position=chr1:69680527-69680549) \| \| 37 \| [4:intergenic:GABRA1-GABRG2](http://genome.ucsc.edu/cgi-bin/hgTracks?db=hg19&position=chr5:161427180-161427202) \| \| 38 \| [4:intergenic:AC005029.1-AC005022.1](http://genome.ucsc.edu/cgi-bin/hgTracks?db=hg19&position=chr7:41128199-41128221) \| \| 39 \| [4:intron:RP4-536B24.3](http://genome.ucsc.edu/cgi-bin/hgTracks?db=hg19&position=chr16:87824727-87824749) \| \| 40 \| [4:intron:SLC25A33](http://genome.ucsc.edu/cgi-bin/hgTracks?db=hg19&position=chr1:9621834-9621856) \| \| 41 \| [4:intron:RBPJ](http://genome.ucsc.edu/cgi-bin/hgTracks?db=hg19&position=chr4:26327025-26327047) \| \| 42 \| [4:intron:ADARB1](http://genome.ucsc.edu/cgi-bin/hgTracks?db=hg19&position=chr21:46629774-46629796) \| \| 43 \| [3:intergenic:LINC00924-RNU2-3P](http://genome.ucsc.edu/cgi-bin/hgTracks?db=hg19&position=chr15:96217302-96217324) \| \| 44 \| [4:intergenic:RGS9-AC006080.1](http://genome.ucsc.edu/cgi-bin/hgTracks?db=hg19&position=chr17:63309549-63309571) \| \| 45 \| [4:intron:SORBS2](http://genome.ucsc.edu/cgi-bin/hgTracks?db=hg19&position=chr4:186789823-186789845) \| \| 46 \| [4:intergenic:AC079117.3-LRRTM4](http://genome.ucsc.edu/cgi-bin/hgTracks?db=hg19&position=chr2:77493703-77493725) \| \| 47 \| [4:intergenic:RP11-159D8.1-RP11-436H16.1](http://genome.ucsc.edu/cgi-bin/hgTracks?db=hg19&position=chr11:38087015-38087037) \| \| 48 \| [4:intergenic:MIR3201-RP11-536P6.3](http://genome.ucsc.edu/cgi-bin/hgTracks?db=hg19&position=chr22:48705097-48705119) \| \| 49 \| [4:intergenic:TRBV30-EPHB6](http://genome.ucsc.edu/cgi-bin/hgTracks?db=hg19&position=chr7:142531385-142531407) \| \| 50 \| [4:intergenic:RP11-124K4.1-AC012175.1](http://genome.ucsc.edu/cgi-bin/hgTracks?db=hg19&position=chr16:5669368-5669390) \| \| 51 \| [4:intergenic:RNY1P3-ATP8A2](http://genome.ucsc.edu/cgi-bin/hgTracks?db=hg19&position=chr13:26533256-26533278) \| \| 52 \| [4:intron:TTLL11](http://genome.ucsc.edu/cgi-bin/hgTracks?db=hg19&position=chr9:124750560-124750582) \| \| 53 \| [4:intron:CTB-129O4.1](http://genome.ucsc.edu/cgi-bin/hgTracks?db=hg19&position=chr5:179720447-179720469) \| \| 54 \| [4:intergenic:RP11-555G19.1-AP003062.1](http://genome.ucsc.edu/cgi-bin/hgTracks?db=hg19&position=chr11:134823756-134823778) \| \| 55 \| [4:intron:GLYR1](http://genome.ucsc.edu/cgi-bin/hgTracks?db=hg19&position=chr16:4875217-4875239) \| \| 56 \| [4:intergenic:KB-1460A1.3-KB-1460A1.5](http://genome.ucsc.edu/cgi-bin/hgTracks?db=hg19&position=chr8:102154604-102154626) \| \| 57 \| [4:intergenic:RP3-510D11.1-H6PD](http://genome.ucsc.edu/cgi-bin/hgTracks?db=hg19&position=chr1:9271508-9271530) \| \| 58 \| [4:intergenic:CTD-2328D6.1-RP1-31B8.1](http://genome.ucsc.edu/cgi-bin/hgTracks?db=hg19&position=chrX:108432850-108432872) \| \| 59 \| [4:intergenic:PRIMA1-FAM181A-AS1](http://genome.ucsc.edu/cgi-bin/hgTracks?db=hg19&position=chr14:94329210-94329232) \| \| 60 \| [4:intergenic:SGCD-CTB-56J15.1](http://genome.ucsc.edu/cgi-bin/hgTracks?db=hg19&position=chr5:155869089-155869111) \| \| 61 \| [4:intron:DOCK1](http://genome.ucsc.edu/cgi-bin/hgTracks?db=hg19&position=chr10:129249069-129249091) \| \| 62 \| [4:intergenic:AC097372.1-LSM6](http://genome.ucsc.edu/cgi-bin/hgTracks?db=hg19&position=chr4:147052835-147052857) \| \| 63 \| [3:intergenic:CTC-786C10.1-COX6CP16](http://genome.ucsc.edu/cgi-bin/hgTracks?db=hg19&position=chr16:85225505-85225527) \| \| 64 \| [4:exon:INPP4A](http://genome.ucsc.edu/cgi-bin/hgTracks?db=hg19&position=chr2:99154410-99154432) \| \| 65 \| [4:intergenic:AC004070.1-RP11-51C14.1](http://genome.ucsc.edu/cgi-bin/hgTracks?db=hg19&position=chrX:139431281-139431303) \| \| 66 \| [4:intergenic:AC005150.1-RP11-580J4.1](http://genome.ucsc.edu/cgi-bin/hgTracks?db=hg19&position=chr4:163780578-163780600) \| \| 67 \| [4:intergenic:RP11-629E24.2-LINC00365](http://genome.ucsc.edu/cgi-bin/hgTracks?db=hg19&position=chr13:30605959-30605981) \| \| 68 \| [4:intergenic:MIR145-CSNK1A1/CTB-89H12.4](http://genome.ucsc.edu/cgi-bin/hgTracks?db=hg19&position=chr5:148848989-148849011) \| \| 69 \| [4:intergenic:RP11-760L24.1-CCBE1](http://genome.ucsc.edu/cgi-bin/hgTracks?db=hg19&position=chr18:57271702-57271724) \| \| 70 \| [4:intergenic:RP11-555H23.1-SNORA20](http://genome.ucsc.edu/cgi-bin/hgTracks?db=hg19&position=chrX:5293711-5293733) \| \| 71 \| [4:intergenic:RP11-410C4.4-MARK1](http://genome.ucsc.edu/cgi-bin/hgTracks?db=hg19&position=chr1:220686120-220686142) \| \| 72 \| [4:intron:TBX5](http://genome.ucsc.edu/cgi-bin/hgTracks?db=hg19&position=chr12:114840830-114840852) \| \| 73 \| [4:intergenic:RP11-745I13.1-RP11-643G5.6/NOX4](http://genome.ucsc.edu/cgi-bin/hgTracks?db=hg19&position=chr11:89245601-89245623) \| \| 74 \| [4:intron:RP11-502M1.2](http://genome.ucsc.edu/cgi-bin/hgTracks?db=hg19&position=chr4:161489641-161489663) \| \| 75 \| [4:intergenic:ST6GAL2-PPP1R2P5](http://genome.ucsc.edu/cgi-bin/hgTracks?db=hg19&position=chr2:107524537-107524559) \| \| 76 \| [4:intron:TGFA](http://genome.ucsc.edu/cgi-bin/hgTracks?db=hg19&position=chr2:70745017-70745039) \| \| 77 \| [4:intergenic:U3-KB-1683C8.1](http://genome.ucsc.edu/cgi-bin/hgTracks?db=hg19&position=chr8:98568057-98568079) \| \| 78 \| [4:intron:RP11-454P21.1](http://genome.ucsc.edu/cgi-bin/hgTracks?db=hg19&position=chr5:17694165-17694187) \| \| 79 \| [4:intergenic:OLFM4-LINC01065](http://genome.ucsc.edu/cgi-bin/hgTracks?db=hg19&position=chr13:53630536-53630558) \| \| 80 \| [4:intergenic:LRRN4-FERMT1](http://genome.ucsc.edu/cgi-bin/hgTracks?db=hg19&position=chr20:6044357-6044379) \| \| 81 \| [4:intergenic:TGFBR3-RN7SL653P](http://genome.ucsc.edu/cgi-bin/hgTracks?db=hg19&position=chr1:92271352-92271374) \| \| 82 \| [4:intron:RAD51B](http://genome.ucsc.edu/cgi-bin/hgTracks?db=hg19&position=chr14:69006189-69006211) \| \| 83 \| [4:exon:CLIC5](http://genome.ucsc.edu/cgi-bin/hgTracks?db=hg19&position=chr6:45882198-45882220) \| \| 84 \| [4:intron:FHOD3](http://genome.ucsc.edu/cgi-bin/hgTracks?db=hg19&position=chr18:34202124-34202146) \| \| 85 \| [4:intron:SETD2](http://genome.ucsc.edu/cgi-bin/hgTracks?db=hg19&position=chr3:47111637-47111659) \| \| 86 \| [4:intergenic:CTD-2215E18.3-CTD-2278B20.1](http://genome.ucsc.edu/cgi-bin/hgTracks?db=hg19&position=chr5:96616313-96616335) \| \| 87 \| [4:intron:FMN1](http://genome.ucsc.edu/cgi-bin/hgTracks?db=hg19&position=chr15:33197566-33197588) \| \| 88 \| [4:intron:LTK](http://genome.ucsc.edu/cgi-bin/hgTracks?db=hg19&position=chr15:41797548-41797570) \| \| 89 \| [4:intron:PAPPA2](http://genome.ucsc.edu/cgi-bin/hgTracks?db=hg19&position=chr1:176646418-176646440) \| \| 90 \| [4:intron:LYRM4](http://genome.ucsc.edu/cgi-bin/hgTracks?db=hg19&position=chr6:5130304-5130326) \| \| 91 \| [4:intergenic:RP11-473C19.1-ANKS1B](http://genome.ucsc.edu/cgi-bin/hgTracks?db=hg19&position=chr12:99418849-99418871) \| \| 92 \| [4:intergenic:RP11-104D21.1-RP11-104D21.2](http://genome.ucsc.edu/cgi-bin/hgTracks?db=hg19&position=chrX:50783852-50783874) \| \| 93 \| [4:intergenic:AL592205.2-DPYD](http://genome.ucsc.edu/cgi-bin/hgTracks?db=hg19&position=chr1:97420116-97420138) \| \| 94 \| [3:intron:BRPF3](http://genome.ucsc.edu/cgi-bin/hgTracks?db=hg19&position=chr6:36178352-36178374) \| \| 95 \| [4:intergenic:UTY-AC006371.1](http://genome.ucsc.edu/cgi-bin/hgTracks?db=hg19&position=chrY:15714327-15714349) \| \| 96 \| [4:intron:AC003664.1](http://genome.ucsc.edu/cgi-bin/hgTracks?db=hg19&position=chr17:13208955-13208977) \| \| 97 \| [3:intron:ACOT12](http://genome.ucsc.edu/cgi-bin/hgTracks?db=hg19&position=chr5:80643886-80643908) \| \| 98 \| [3:intergenic:RP13-143G15.4-PDE7B](http://genome.ucsc.edu/cgi-bin/hgTracks?db=hg19&position=chr6:136427713-136427735) \| \| 99 \| [3:intron:NGF](http://genome.ucsc.edu/cgi-bin/hgTracks?db=hg19&position=chr1:115866273-115866295) \| \| 100 \| [4:intron:RP3-429O6.1](http://genome.ucsc.edu/cgi-bin/hgTracks?db=hg19&position=chr6:6906732-6906754) \| \| 101 \| [4:intergenic:SNORD74-RNA5SP371](http://genome.ucsc.edu/cgi-bin/hgTracks?db=hg19&position=chr12:107875715-107875737) \| \| 102 \| [4:intergenic:ILDR1-Y_RNA](http://genome.ucsc.edu/cgi-bin/hgTracks?db=hg19&position=chr3:121742705-121742727) \| \| 103 \| [4:intron:PCCA](http://genome.ucsc.edu/cgi-bin/hgTracks?db=hg19&position=chr13:101176221-101176243) \| \| 104 \| [4:intron:RP11-526A4.1](http://genome.ucsc.edu/cgi-bin/hgTracks?db=hg19&position=chr4:150500100-150500122) \| \| 105 \| [4:intergenic:NUDCD3-AC004453.8](http://genome.ucsc.edu/cgi-bin/hgTracks?db=hg19&position=chr7:44479690-44479712) \| \| 106 \| [3:intergenic:CTD-2572N17.1-CTD-2537L20.1](http://genome.ucsc.edu/cgi-bin/hgTracks?db=hg19&position=chr11:42349998-42350020) \| |  |  |  |  |
|  |  |  |  |  |
|  |  |  |  |  |
|  |  |  |  |  |
|  |  |  |  |  |
|  | | | | |
